# Malignant pleural mesothelioma

**DOI:** 10.4103/0970-2113.48900

**Published:** 2009

**Authors:** Sukhesh Rao

**Affiliations:** *Department of Tuberculosis and Respiratory Diseases, Yenepoya Medical College, Mangalore-575 018, Karnataka, India*

**Keywords:** Mesothelioma, pleural effusion, abestos

## Abstract

Malignant mesothelioma is one of the rare tumors of pleura. One such case in a 57-year-old male, who presented with hemorrhagic pleural effusion and had no history of asbestos exposure, is reported here. The rarity, unusual presentation, and implications are discussed.

## INTRODUCTION

Malignant pleural mesothelioma (MPM) is the most common neoplasm of pleura.^1^ It is a cancerous proliferation of mesothelial cells that involves a large extent of pleural cavity.^2^ A strong etiological correlation with asbestos exposure is well proven.^1^,^3^ Unusual presentations are also reported, though rarely.^4^ We report here one such case.

## CASE HISTORY

Mr. P, a 57-year-old male, presented with complaints of dyspnea on exertion and left-sided chest pain for 15 days. History revealed smoking index to be 250. He was a known diabetic under regular treatment. There was no other significant history.

Examination revealed thin built, anemic, dyspneic individual with signs of pleural effusion on the left side. On investigation, chest X-ray revealed left-sided pleural effusion. There was no other pleural or parenchymal abnormality. Diabetes was under adequate control. Other hematological and biochemical parameters were within normal limits.

**Figure 1:**
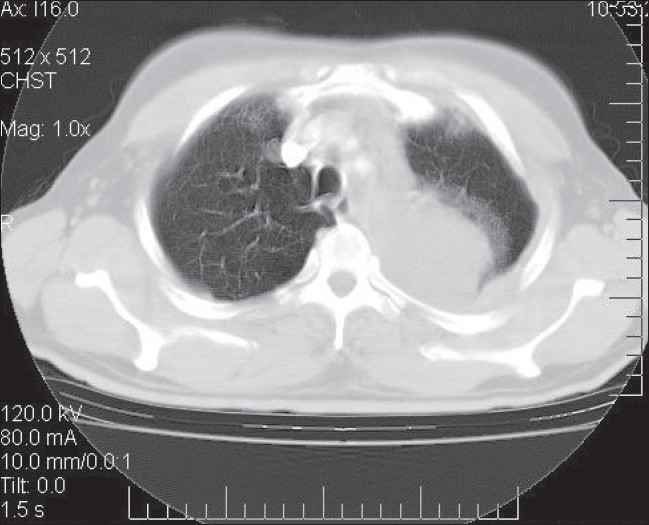
CT scan showing opaque left hemithorax with pleural effusion

**Figure 2:**
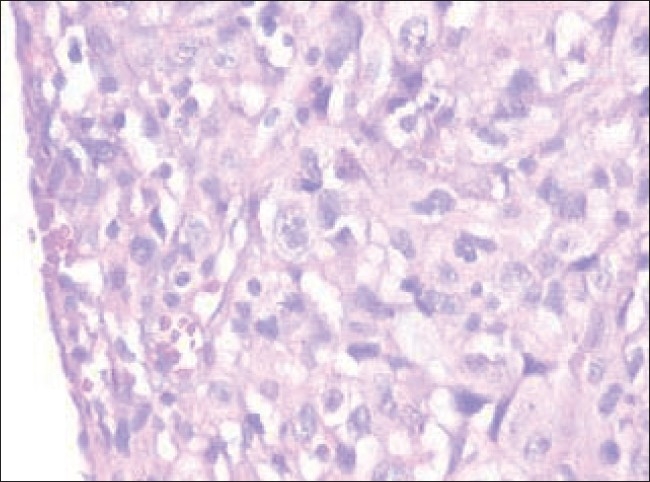
Microscopic appearance which shows neoplastic cells arranged in solid nests, cytologic atypia, and hyperchromatic nuclei suggestive of mesothelioma (H and E stain; ×40)

Pleural aspiration revealed hemorrhagic fluid which was exudative. Fluid examination did not show acid fast bacilli or any malignant or abnormal cells. Hence, CT scan of thorax was perfomed that suggested the lesions to be probably infective, with no evidence of malignancy [Figure 1]. Empirically, he was put on antitubercular treatment. On the fourth day he developed acute severe dyspnea. Examination revealed massive effusion. Intercostal drainage was instituted and the fluid was once again examined; but it did not reveal malignant cells even on three consecutive occasions. Finally, a thoracotomy and open lung biopsy was done which confirmed the diagnosis of mesothelioma [Figure 2].

## DISCUSSION

MPM is a rare tumor even in Western world and still rarer in India. The incidence in men ranges from 7–13 per million per year. In population unexposed to asbestos it is still rarer, with reported incidence of 1–2 per million per year.^5^,^6^

MPM usually occurs in males with a male to female ratio of 2.6:1. It is usually related to asbestos exposure, though rarely it can occur in patients not exposed to asbestos. In such cases, the postulated correlation is operation of other carcinogens, genetic factors, and viral infections.^7^

Patients usually present with pleural effusions. Radiographic investigations reveal pleural effusion (exudative/hemorrhagic), pleural nodular shadows (diffuse or localized), or involvement of lungs, ribs, spine, etc.^8^ Pleural fluid cytology may sometimes reveal the diagnosis, but usually definitive diagnosis is based on histological evidence on examination of pleural tissue.

Histologically, MPM are of three types: (a) sarcomatoid type, appearing as a spindle cell carcinoma resembling fibrosarcoma, (b) epithelial type, consisting of cuboidal, columnar, or flattened cells forming a tubular and papillary structure resembling adenocarcinoma, and (c) biphasic type, containing both epithelial and sarcomatoid (mixed) patterns. The histological picture in this case was suggestive of epithelial type.

A search of literature for reported cases from India confirmed the rarity. In a retrospective study,^9^ only 15 cases were reported over a 25-year period. Another study^2^ reported only three cases over a 10-year period. Whether this is because of lesser number of cases, or lesser awareness and diagnosis, is difficult to predict.

The rarity of the occurrence, the absence of exposure to asbestos, and the unusual presentation prompted us to report this case.

## References

[1] Patel SN, Kettner NW (2005). Malignant pleural mesothelioma: A case report. J Manipulative Physiol Ther.

[2] Nadgouda UG, Soppimath SS, Datta KS, Shiggaon UN, Babu KR (2001). Malignant pleural mesothelioma. J Assoc Physicians India.

[3] Bruce WS, Robinson MD, Lake RA (2005). Advances in malignant mesothelioma. N Engl J Med.

[4] Kashyap AS, Kashyap S (2001). An elderly man with pleural effusion and abnormal behaviour. Postgrad Med J.

[5] Miller BH, Rosado-de-Christenson, Mason AC, Fleming MV, White CC, Krasna MJ (1996). Malignant pleural mesothelioma: Radiologic-pathologic correlation. Radiographics.

[6] Hansen J, Klerk NH, Musk AW, Hobbs MS (1998). Environmental exposure to crociodolite and mesothelioma: Exposure response relationship. Am J Respir Crit Care Med.

[7] Fraser RC, Colman N, Pare PA (2006). Synopsis of diseases of chest.

[8] Wang ZJ, Reddy GP, Gotway MB, Higgins CB, Jablons DM, Ramaswamy M (2004). Malignant pleural mesothelioma: Evaluation with CT, MR imaging and PET. Radiographics.

[9] Kini U, Shariff S, Thomas JA (1992). Primary pleural mesothelioma in S. India: A 25 year study. J Surg Oncol.

